# Healthcare accessibility to rabies post-exposure prophylaxis in rural Kenya: implications for vaccine placement and travel time

**DOI:** 10.3389/fmicb.2026.1703736

**Published:** 2026-02-04

**Authors:** Mumbua Mutunga, Mutono Nyamai, Stella Mazeri, Daniel Ksee, Marybeth Maritim, Chuchu Mbaire, Katie Hampson, S. M. Thumbi

**Affiliations:** 1 Centre for Epidemiological Modelling and Analysis, University of Nairobi, Nairobi, Kenya; 2 Institute of Tropical and Infectious Diseases, University of Nairobi, Nairobi, Kenya; 3 Paul G. Allen School for Global Health, Washington State University, Pullman, WA, United States; 4 Easter Bush Veterinary Centre, The University of Edinburgh, Edinburgh, United Kingdom; 5 Department of Agriculture, Irrigation, Livestock, and Fisheries Development, County Government of Makueni, Wote, Makueni, Kenya; 6 Department of Clinical Medicine and Therapeutics, University of Nairobi, Nairobi, Kenya; 7 Institute of Biodiversity, Animal Health & Comparative Medicine, University of Glasgow, Glasgow, United Kingdom; 8 Institute of Immunology and Infection Research, School of Biological Sciences, University of Edinburgh, Edinburgh, United Kingdom

**Keywords:** healthcare, Kenya, post exposure prophylaxis, rabies, spatial accessibility, travel time, vaccines

## Abstract

**Background:**

Rabid animal bites are a medical emergency, requiring immediate post-exposure prophylaxis (PEP) to prevent human deaths. This study modeled geographical accessibility to health facilities stocking rabies vaccines and assessed the impact of optimizing vaccine placement within a rural Kenyan healthcare network.

**Methods:**

We used geocoordinates of Kenyan health facilities from an open-access inventory and identified those stocking PEP in Makueni County. Using population distribution data and a travel time friction surface, we estimated travel times to all health facilities, including those stocking PEP. We assessed the proportion of the population within 30 min, 1, 1.5, 2, and >2 h of these facilities. We further used dog-bite data from contact-tracing studies (2017–2021) to estimate the shortest distance and travel time for patients accessing PEP.

**Results:**

Two-thirds (66.6%) of the population lived within a 30-min walk to any health facility, but only 7.4% had similar access to a PEP facility. Using the non-motorized travel scenario, 66.6, 29.4, 3.5, 0.3, and 0.2% of the population were within 30 min, 1 h, 1.5 h, 2 h, and more than 2 h of walking time, respectively, to any health facility. Among 931 bite patients identified through contact tracing, 376 (40.4%) could reach any health facility within 10 min, while only 289 (35.4%) had similar access to a PEP facility. Additionally, 27 (2.9%) required over 60 min to reach any health facility, while 26 (3.2%) needed over 60 min specifically to access PEP. When considering motorized travel, the entire population was within 30 min of both any facility and a PEP facility.

**Conclusion:**

Our findings demonstrate that increased geographic distance and longer travel times to health facilities substantially reduce accessibility to rabies PEP, particularly in rural settings where reliance on non-motorized travel is common. To our knowledge, this is the first study to provide quantitative, county-specific estimates of travel time impacts on PEP access in rural Kenya, filling a critical evidence gap for planning PEP distribution under Kenya’s Stepwise Approach to Rabies Elimination (SARE). Optimizing the placement of PEP within existing healthcare networks can increase the proportion of the population able to reach services within recommended time thresholds. Expanding vaccine availability at strategically located facilities would therefore improve timely PEP to bite victims and support efforts to prevent human rabies deaths. These results highlight the importance of incorporating geographic accessibility analyses into planning for rabies elimination programs.

## 1 Introduction

Rabies is a neglected zoonotic disease estimated to kill 59,000 people annually, mainly in Africa and Asia (Hampson et al., 2015). Transmission to humans is nearly always through bites from rabid domestic dogs, which serve as the main reservoir for the virus (Lembo et al., 2008; Nadal et al., 2023).

Dog-mediated rabies has been controlled as a public health problem in many middle-income countries and eliminated from high-income countries through a combination of mass dog vaccination, effective management of free-roaming dog populations, and the prompt provision of post-exposure prophylaxis (PEP) to persons exposed to the virus (Cleaveland et al., 2018; Fooks et al., 2017). The global strategy to reach the target of zero human deaths by dog-mediated rabies by 2030 hinges on these two approaches, and additional efforts to improve surveillance of human and animal rabies, and community education and awareness of rabies (Bitek et al., 2019; Minghui et al., 2018; World Health Organization Food and Agriculture Organization of the United Nations World Organisation for Animal Health Global Alliance for Rabies Control, 2018).

Once a person is bitten by a suspected rabid dog, the first prevention measure should be to thoroughly clean the wound with soap and water and then seek medical attention. PEP involves wound cleaning and immediate post-exposure vaccination and, when indicated, includes the recommended infiltration of rabies immunoglobulin (RIG) into the wound(s) (Warrell, 2012). However, RIG often has limited availability, and therefore, WHO recommends that in such circumstances, RIG is targeted to WHO Category III patients considered to be high risk, where exposures involve multiple wounds, deep bites, and/or bites to highly innervated parts of the body (Nadal et al., 2023). PEP prevents the onset of clinical disease but access remains a challenge, particularly in rural areas (WHO Rabies Modelling Consortium, 2019). For the purposes of this study, PEP will refer to rabies post-exposure vaccination as RIG was largely unavailable.

In many rabies endemic countries in Africa and Asia, the provision of rabies PEP suffers from access, procurement, and distribution challenges. The main issues include the high costs of both the RIG and vaccines, their availability in only a limited subset of health facilities, frequent stockouts, inadequate systems for forecasting demand and distribution, the inability of bite patients to afford PEP, and low awareness of rabies among community members and healthcare workers regarding appropriate actions for rabies prevention (Changalucha et al., 2019; Chuchu et al., 2022; Sreenivasan et al., 2019; Wambura et al., 2019).

In Kenya, rabies remains endemic and continues to pose a major public health burden. Recent national surveillance data indicate an average of 53 clinically reported human rabies cases and 6 human deaths per month, equivalent to approximately 13 cases per million people annually, although the true burden is estimated at approximately 2,000 deaths per year due to substantial underreporting (Kahariri et al., 2025; Zoonotic Disease Unit, 2014). Surveillance in animals reveals sustained dog rabies transmission, with an estimated 0.17 dog rabies cases per 1,000 dogs per year nationally. Makueni County is among Kenya’s designated rabies elimination pilot counties and continues to report suspected rabid dogs and frequent bite exposures, consistent with ongoing endemic transmission. Dog vaccination coverage in rural Kenyan counties often remains below the 70% threshold required to interrupt transmission, and challenges in timely access to PEP contribute to persistent human risk. These epidemiological patterns underline the importance of improving geographical access to PEP in rural, high-risk settings such as Makueni.

In 2018, Gavi, the Vaccine Alliance, made a commitment to invest in human rabies vaccines to improve their access across endemic countries that qualify for Gavi support (GAVI, 2018). This investment aligns with the Zero-by-30 goal. Improving access to PEP by removing cost barriers to patients and increasing access to these vaccines has been argued to not only be a critical necessity for ending human deaths from rabies but also a social justice issue (WHO Rabies Modelling Consortium, 2019; Thumbi et al., 2022; Wentworth et al., 2019). PEP is an on-demand vaccine with expensive purchase costs, intermittent demand, and consecutive doses spread over time. This makes it uneconomical and unfeasible to stock it in all the health facilities and instead requires optimally placing this life-saving vaccine in health facilities guided by the population at risk, rabies incidence, and vaccine demand.

Travel time has been reported as a notable barrier to healthcare. This is paramount in time-bound emergencies like rabies, where delays in accessing care may lead to deaths. Only 33.3% of African countries are reported to have more than 80% of the population located within a 2-h travel time of emergency care (Ouma et al., 2018). The distance and driving time to a health facility have been shown to be consistently and significantly associated with severe malaria in Yemen (Al-Taiar et al., 2008). Similarly, pregnant women were found to only be willing to travel a distance of 2 km while seeking care; otherwise, they preferred a home delivery (Mwaliko et al., 2014). These studies highlight how physical distance, transport, and financial constraints can be major barriers to seeking care.

Spatial accessibility analyses have been used to estimate the proportion of the population within a defined travel time to health facilities, including for hospital care, surgery, and emergency services, such as treatment of snake envenoming (Ouma et al., 2018; Broer et al., 2018; Ochoa et al., 2023). For rabies, studies have used geospatial analysis to estimate the impact of improved PEP access and targeting interventions, including where health facilities should be set up to provide PEP (Baron et al., 2022; Rajeev et al., 2021). Physical accessibility plays a major role on whether bite patients will be able to seek care, especially considering the repeat doses required as part of post-exposure vaccination regimens, for example, the updated Essen regimen is spread over a one-month period with one (1 mL) dose of vaccine administered intramuscularly (IM) on day 0, 3, and 7, and a fourth dose between day 14 and 28 following exposure (World Health Organization, 2018). Geographical distance to facilities and the time required for seeking care are important determinants of access. Placement is crucial and could translate to lives saved.

In Kenya, the Stepwise Approach to Rabies Elimination (SARE) was adopted, and a strategic plan was developed to gradually reduce the risk of dog-mediated rabies in humans by 2030 (Bitek et al., 2019). In this study, we focus on Makueni County, a rural region in Kenya that is endemic for rabies and serves as a national rabies elimination pilot site, and we adopt time thresholds used in emergency services to estimate the proportion of the population with access to PEP. While rural healthcare access disparities are recognized, this study provides the first quantitative, county-specific estimates of travel time impacts on PEP access in this setting, which are essential for planning PEP distribution under Kenya’s SARE program and Gavi-supported expansion. We estimated travel times to access PEP, considering both motorized and non-motorized (walking) travel to any health facility and to currently designated PEP facilities. From contact tracing, we also estimated the time and distance that bite patients traveled to seek PEP.

## 2 Materials and methods

**Figure 1:**
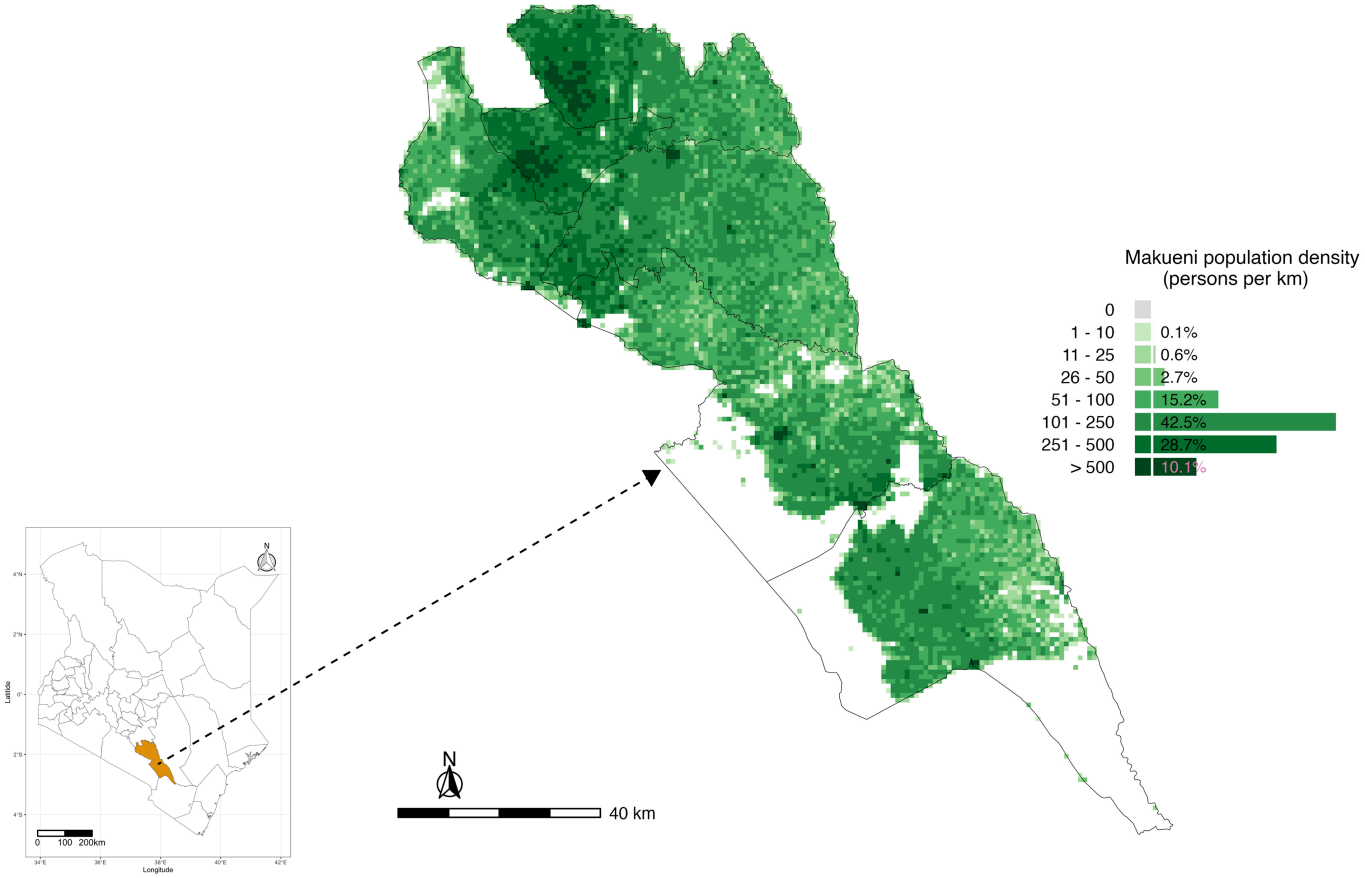
The study population of Makueni County, Kenya, and its six administrative sub-counties. Sources: WorldPop, Ministry of Health, and the Database of Global Administrative Areas (GADM, http://www.gadm.org/).

We carried out this study in Makueni County, a rabies endemic region in Kenya implementing the national rabies elimination program (Bitek et al., 2019). Makueni was one of the rabies elimination pilot counties in Kenya, strategically selected due to its high disease burden (Bitek et al., 2019). It is also one of the counties that was selected to implement the SARE rabies elimination approach in Kenya when the strategy was launched. The 2019 Kenya Population Census reported the county to have a population of 987,653, and a population density of 121 people/km^2^ (Kenya National Bureau of Statistics, 2019). Figure 1 shows the location of Makueni County, its six sub-counties and the population density across the county. Among the 47 counties in Kenya, Makueni pioneered subsidized healthcare to increase access to services for its population through the universal healthcare coverage program that started in 2016 (Njeru et al., 2019).

### 2.1 Data sources

#### 2.1.1 Healthcare facilities

We obtained data on all health facilities in Makueni County including their geolocations from the Kenya Master Health Facility List maintained by Kenya’s Ministry of Health, and the published spatial database of public health facilities in sub-Saharan Africa (Moturi et al., 2022). For the facilities that had not been georeferenced, we used Google Earth to find the address geocoding of the place names. The databases gave a total of 391 healthcare facilities in Makueni County, with 252 public facilities (64%), 32 faith-based (8%), 5 associated with non-governmental organizations (1%), and 102 private facilities (26%). Of the 252 public facilities, only 12 (5%) are supported by the county government to stock rabies PEP. Current availability of RIG was not assessed as part of the study; instead, we focused on the rabies vaccine.

The 2014–2030 Kenya Health Policy organizes Kenya Health System into four tiers: level 1: community health units; level 2: primary care facilities comprising of dispensaries, clinics, and health centers; level 3: county hospitals comprising primary and secondary care hospitals; level 4: national referral hospitals comprising of tertiary care hospitals (Kenya Ministry of Health, 2010). The estimated population falling within the catchment areas for facilities under each level is estimated as 5,000, 10,000–30,000, 100,000–500,000, and 1,000,000 for levels 1, 2, 3, and 4, respectively (Ministry of Health Kenya, 2020).

**Figure 2:**
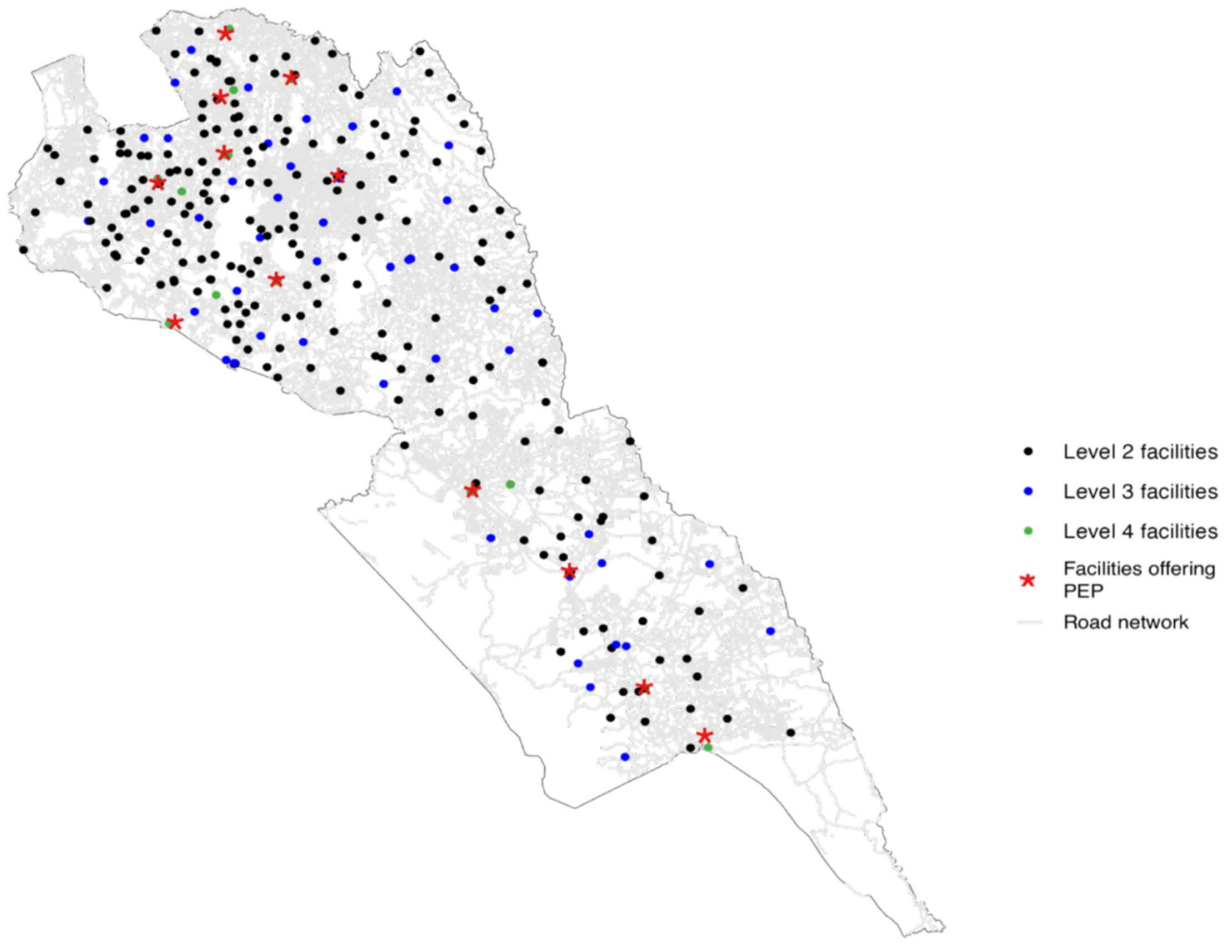
Road network coverage and locations of health facilities in Makueni County, Kenya. Source: OpenStreetMap, Ministry of Health. Shapefile source: Database of Global Administrative Areas (GADM, http://www.gadm.org/).

Facilities that stock PEP in Makueni consist of the county referral hospital and sub-county health facilities across all six sub-counties. Three strategic factors were considered by the county government when placing PEP in these 12 facilities. First, the expensive nature of the vaccine meant that stocking every facility was not feasible, second the storage needs of the vaccine, and finally, the skill of the personnel trained to handle the vaccine was mostly found in these facilities. Figure 2 shows the location of all health facilities in Makueni by level and the facilities stocking PEP in relation to the road network.

#### 2.1.2 Population data

We obtained estimates of the population in Makueni County at 100 by 100 m spatial resolution from WorldPop (Lloyd et al., 2019). We extracted the Makueni population dataset from the 2019 WorldPop spatial distribution of constrained population density dataset. This dataset is built on the 2019 census data with additional datasets including administrative maps, road network maps, and settlement maps to estimate populations at sub-location level and redistributed to the 100 m^2^ resolution grid cells (Stevens et al., 2015).

#### 2.1.3 Road network data

The road network data for Makueni were obtained from publicly available data from OpenStreetMap. These data contained information on roads that allowed for both motorized and non-motorized travel; major roads that comprised primary, secondary, and tertiary roads; minor roads including smaller roads and roads in residential areas; very small roads composed of tracks in forests that are used to access shelters and gardens; and paths unsuitable for cars but used as footpaths and for cycling. Approximately 67% of roads in Makueni are classified as residential or unclassified minor roads, which typically experience low traffic volumes. As a result, traffic congestion was not considered a major factor influencing travel time to health facilities and was therefore excluded from the accessibility analysis (Figure 2).

#### 2.1.4 Friction layer

We adopted the MalariaAtlas friction layer that provided the estimates of time travel (in min) to the nearest health facility in Makueni by motorized transport and non-motorized transport (walking). We analyzed accessibility looking at the entire healthcare system (all the facilities regardless of the ownership/type) as well as at just PEP stocking facilities. The methodology behind the MalariaAtlas has been described in length elsewhere (Weiss et al., 2020; Weiss et al., 2018). Non-motorized transport is primarily used to access motorized transport, especially in rural settings such as Makueni, where many roads are unclassified and residential. To address this, we modeled both scenarios: non-motorized travel times to roads that facilitate motorized travel and travel times to health facilities by either motorized or non-motorized means. This approach ensures a comprehensive understanding of accessibility challenges in these areas.

#### 2.1.5 Contact tracing

We carried out contact tracing of bite patients that presented at the hospitals within Makueni County from 2017 to 2018 and 2020–2021. These data comprised geolocations of the homesteads of patients who accessed PEP and the facilities they attended. The contact tracing data were collected as part of a risk assessment exercise to ensure that the biting dog was indeed rabid or not. This was also to make sure that anyone who may have had an encounter with a suspected rabid animal and did not visit the facility for PEP seeks treatment as well. During the contact tracing exercise, a questionnaire would be administered to understand the circumstance in which the bite occurred. In this study, “contact tracing” referred to a follow-up investigation conducted after a reported bite incident. During this process, a structured questionnaire was administered to the bite victim or witness to document the circumstances of the bite, the behavior of the biting animal before and after the incident, and whether the animal had died, disappeared, or exhibited clinical signs consistent with rabies. These epidemiological criteria were used to classify the biting animal as a suspected rabid dog. We were also interested to find out the name of the facility they sought for PEP, and in case they were referred, what was the name of the referral facility. This information was important so that we could calculate the time and distance traveled seeking the rabies vaccine.

### 2.2 Accessibility analysis

Geographical accessibility modeling entailed combining distance and time metrics to measure the ease or difficulty of an individual in accessing a facility, resource, or service. Health facility placement should incorporate capacity and ease in accessibility, while seeking medical care. A health facility offering PEP should be easily accessible to the population at risk and have a constant supply of the rabies vaccine. We considered the ideal situation whereby the Makueni population accessed facilities using transportation at the fastest speeds (motorized mode) and the worst situation whereby the population accessed the facilities on foot and the lowest speed of transport.

We used the R programming language to determine the proportion of the population in Makueni County within 30 min, 1 h, 1.5 h, 2 h, and >2 h to two scenarios: to any health facility and to the 12 health facilities that are currently supported by the county government to stock PEP. This analysis was important to determine whether the entire population is within a 2-h time travel of a PEP stocking health facility, which would encourage bite patients to complete subsequent rabies doses.

We also calculated the time and distances that the contract-traced patients covered to travel to the facility that they visited for PEP. In cases where a patient was referred for PEP, we calculated both the time required to reach the initial facility visited and the time to reach the referral facility from the patient’s homestead. We used the distVincentySphere function in the geosphere package in R to calculate the shortest distance between the patients’ residences and the health facilities (Hijmans, 2022).

**Figure 3:**
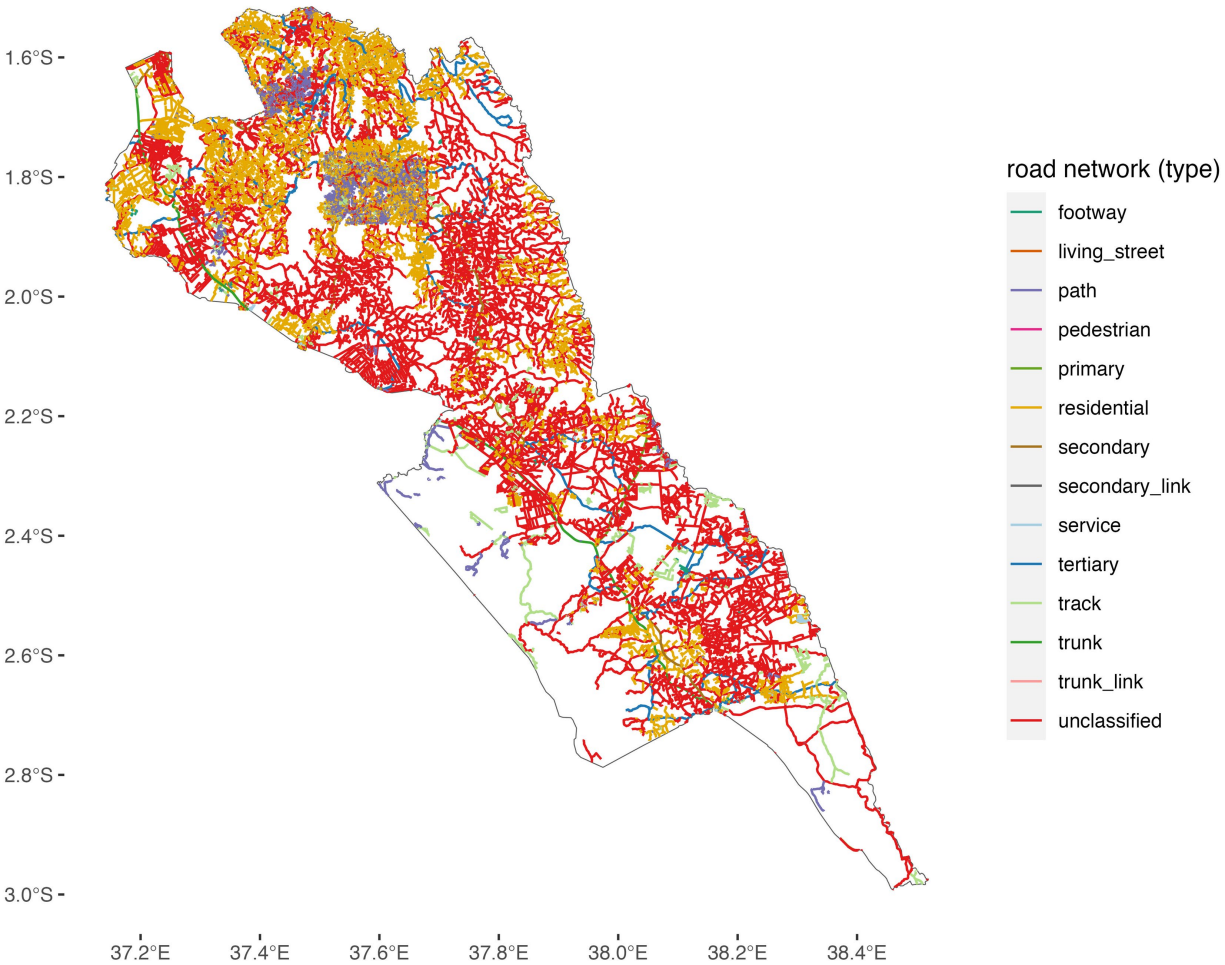
Road network coverage in Makueni County.

The Vincenty’s formulae were used to calculate the distance between the health facilities and the patient’s home. These calculations are mostly used in navigation and are not specifically designed for non-motorized (walking) or motorized (driving); instead, they provide an accurate method for determining the shortest distance between two points on a curved surface (earth), regardless of the mode of transportation. When the average speed is incorporated, then one can calculate the time taken to reach the health facilities. We adopted a speed of 40 km/h from because a majority of the roads in Makueni County were uncategorized (Weiss et al., 2018) (Figure 3).

## 3 Results

### 3.1 Non-motorized scenario

**Figure 4:**
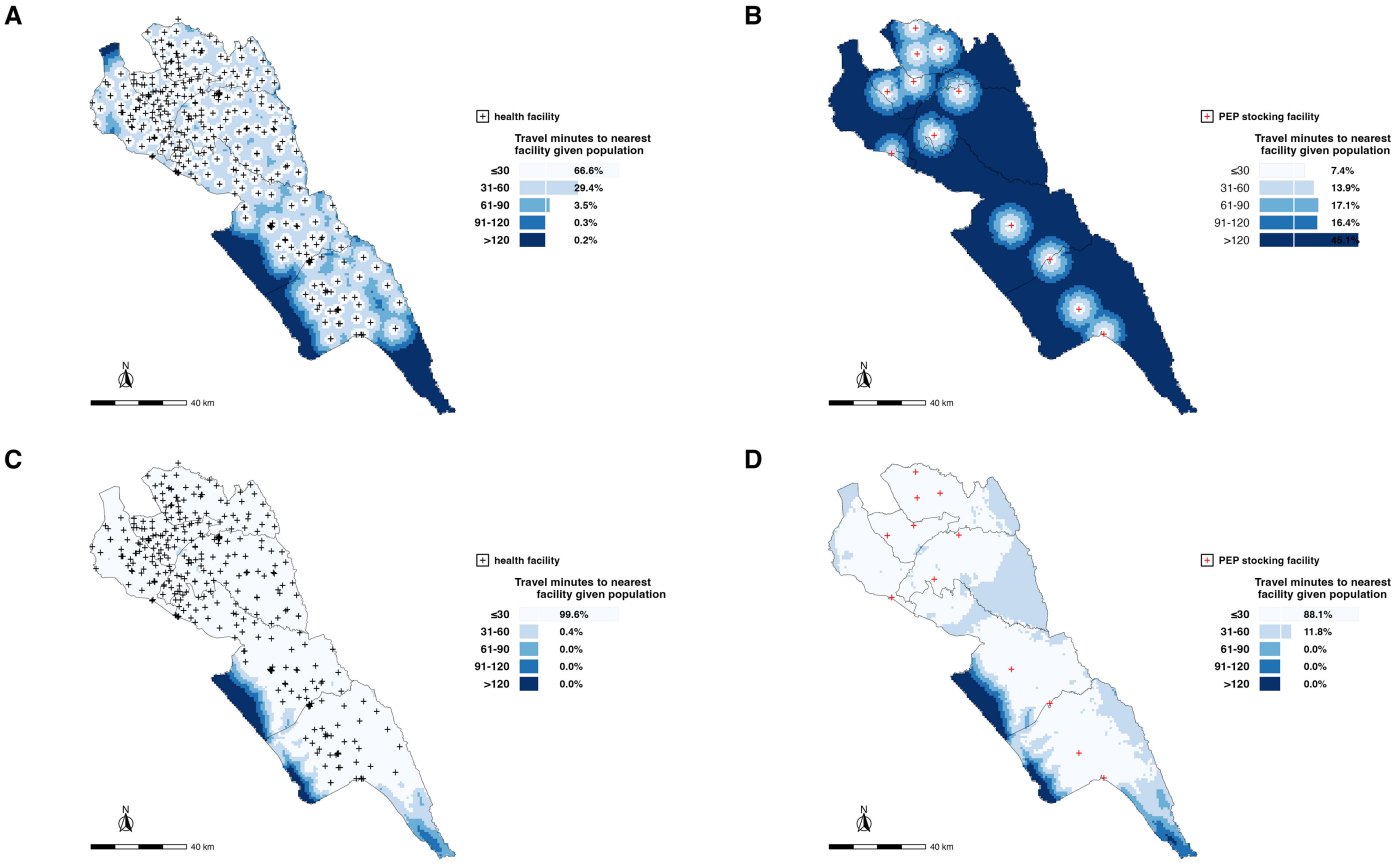
Spatial distribution of health facilities and accessibility to PEP in Makueni County. **(A)** Locations of all health facilities (black dots) with non-motorized travel times to the nearest facility categorized by population accessibility within 30 min, 1 h, 1.5 h, 2 h, and more than 2 h. **(B)** Locations of PEP-stocking facilities (red dots) with non-motorized travel times to the nearest PEP facility, highlighting population access gaps. **(C)** Accessibility to all health facilities for the general population, emphasizing coverage with respect to motorized times. **(D)** Accessibility to PEP-stocking facilities based on motorized travel times, showing areas with significant gaps in timely access. These maps underscore the geographic and logistical challenges in ensuring equitable access to healthcare and life-saving PEP in rural settings.

Considering the 390 geolocated facilities, we estimated that 66.6, 29.4, 3.5, 0.3, and 0.2% of the total population in Makueni were within 30 min, 1 h, 1.5 h, 2 h, and >2 h, respectively, walking time to a health facility (Figure 4A). However, only 7.4% of the population was within 30-min walk to a PEP stocking health facility with more of the population (45.1%) having a walking time of >2 h (Figure 4B).

### 3.2 Motorized scenario

Using geolocations of all 390 health facilities in the county, we estimated that 99.6% of the total population is within 30-min motorized time travel to a health facility within the county (Figure 4C). This reduced to 88.1% of the population being within a 30-min time travel to the 12 PEP stocking facilities supported by the county government (Figure 4D). The entire population was within a one-h time travel to any facility and a PEP stocking facility (Figure 4).

### 3.3 Contact tracing

**Figure 5:**
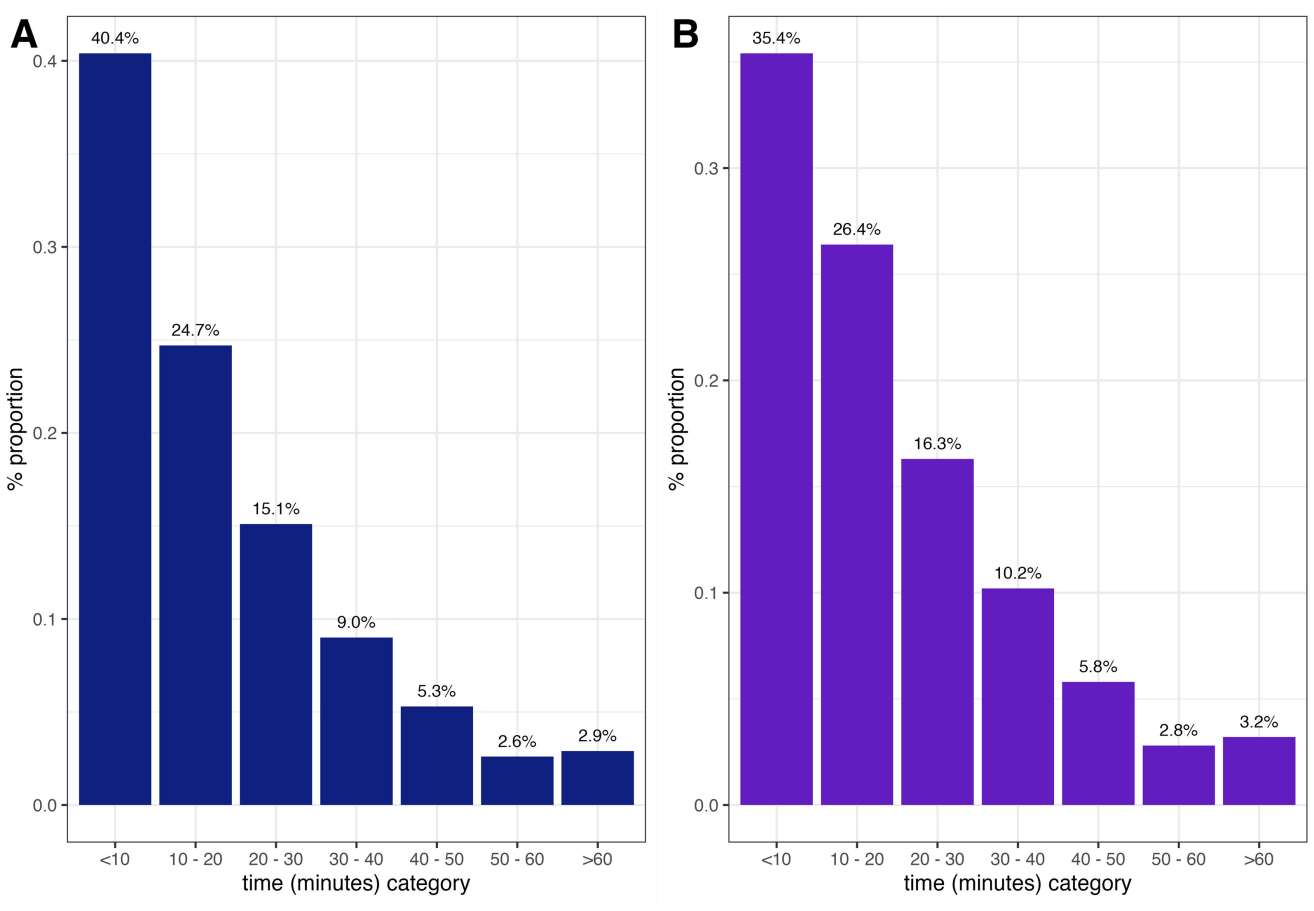
Time taken by bite patients to reach: all healthcare facilities **(A)** vs. PEP facilities only **(B)**.

Given that the distVincentySphere function gives results of the distances between two geographical locations and does not account for roads, transportation modes, or specific routes used by motorized or non-motorized vehicles, we incorporated speed of travel and estimated time taken to move between the two points in this case between the homestead and the health facility seeking PEP. It is important to highlight that in our analysis, we assumed direct access to the facility. In reality, patients often travel to multiple facilities before successfully accessing PEP due to stock-outs or other access barriers. Of the 931 traced patients, 376 (40.4%) were <10 min from the facility where they went to seek PEP treatment, whereas 27 (2.9%) of the patients were >60 min to access PEP treatment (Figure 5A). Of the 931 patients, only 817 lived within the 12 PEP-stocking facilities, 289 (35.4%) were <10 min from any PEP-stocking facilities where they could possibly get PEP, whereas 26 (3.2%) of the patients traced needed >60 min travel time to access PEP treatment (Figure 5B).

## 4 Discussion

In this study, we produced geographical accessibility maps of all the health facilities and PEP stocking facilities using two travel modes (motorized and non-motorized/walking modes), identifying disparities in rural settings that could have low accessibility to lifesaving rabies PEP vaccines. Our results show that although approximately all (99.6 and 88.1%) of the population is within 30 min of any health facility and a PEP stocking facility, respectively, using motorized travel, the same is not true for non-motorized. Makueni is a rural setting where a majority of the population might not necessarily have the financial capacity to use motorized transportation to complete the multiple PEP vaccine doses required, so may either rely on walking to health facilities or forfeit the rabies vaccine. Our results provide valuable information to guide the basic management and targeted placement of specialized healthcare in Makueni County, Kenya.

Accessibility analyses for specific healthcare services are important for understanding how the location of individuals relates to ease and barriers of access to health services. This is especially important for emergency services whose outcomes can be severe in cases where these services are not accessed in time (Ouma et al., 2018; Broer et al., 2018; Chen et al., 2017). Exposure to the rabies virus constitutes a medical emergency that requires immediate injection with PEP vaccines to prevent clinical disease and death (World Health Organization, 2018).

The majority of deaths due to rabies are within rural areas in Africa and Asia and are associated with barriers of access, including high costs of vaccines making them unaffordable for patients, or unavailable when governments place them in only a few health facilities with frequent stockouts (Changalucha et al., 2019; Sreenivasan et al., 2019; Wambura et al., 2019; Knobel et al., 2005). Nonetheless, studies that investigate geographical accessibility (which can be influenced by distances to facilities, methods of transport, roads, and topographies) to these life-saving vaccines are rare. Rabies vaccines also require multiple doses (multiple visits to health facilities), increasing the risk of non-compliance where geographical barriers exist. The few studies of geographical accessibility for rabies PEP report an inverse relationship with increasing travel times to health facilities associated with notable reductions in rabies deaths as PEP is expanded across the health facility network (Baron et al., 2022; Rajeev et al., 2021).

The distribution of travel times to the nearest facility for post-exposure prophylaxis (PEP) highlights significant disparities in access to care. Although the majority had relatively quick access, the longer travel times experienced by some patients, particularly in rural or underserved areas, pose a serious challenge given the time-sensitive nature of PEP. Delays increase the risk of adverse outcomes, emphasizing the need to address geographic and infrastructural barriers, such as improving rural health systems and road networks. These results also raise concerns about equity, as rural populations may face disproportionate challenges, including traveling to multiple facilities due to stock-outs or limited service availability. Understanding the relationship between travel times, PEP access, and treatment completion is crucial, as longer travel times may hinder adherence to the multi-dose PEP regimen. Targeted interventions to improve accessibility, ensure consistent availability of PEP, and promote timely treatment are essential to reducing disparities and improving health outcomes in rabies prevention. Future research into PEP completion rates would also be beneficial, as we were unable to systematically track these in our study.

Digital innovations may also offer promising avenues for strengthening PEP access and adherence when embedded within existing health system platforms. Although our study did not assess the use of digital tools, Kenya’s high mobile penetration and near-nationwide rollout of the community health program provide an opportunity to support timely care-seeking and completion of the multi-dose PEP schedule through established systems. Evidence from rural Kenya shows that SMS reminders delivered via community-based mechanisms can significantly improve PEP compliance among bite victims. Rather than relying on stand-alone, disease-specific applications, which may face sustainability challenges, future efforts could leverage the electronic Community Health Information System (eCHIS) to support bite reporting, referral, follow-up, and dissemination of information on nearby PEP-providing facilities. Integrating rabies exposure notification and PEP tracking into routine community health workflows could strengthen surveillance and adherence while complementing geographical accessibility improvements and aligning with Kenya’s long-term digital health strategy (Chuchu et al., 2023; ZeroRabiesApp-Nigeria, n.d.).

The results of our analysis are insightful for several reasons. First, they highlight the potential inadequacy of the health system, whereby health facilities are within reach for a majority of the population but lack the necessary services or commodities that are lifesaving. Second, we demonstrate that data-driven optimization can lead to the same number of resources (e.g., number of health facilities offering a specific healthcare service or commodity) being accessible to a larger proportion of the population. This concept is likely to be increasingly important as countries shift to universal health coverage and increasing investments in preventative (primary) healthcare (Flores et al., 2021; Gu et al., 2010). Third, improving access to PEP was identified as a critical addition to mass dog vaccinations in ending deaths to human rabies and led to Gavi’s commitment to investing in PEP (WHO Rabies Modelling Consortium, 2019; Thumbi et al., 2022). This type of analysis provides a framework that a country or a region could use for designing programs to increase access to PEP for populations at risk of rabies exposure.

Our accessibility analysis has several limitations. Data on catchment areas for the health facilities were based on the estimated number of persons that each level of health facility was expected to serve. Higher resolution data on the catchment areas of the health facilities in the study area would give improved estimates of the population within defined travel times, while validation of publicly available datasets (WorldPop, OpenStreetMap) in rural Kenyan counties would provide confidence for extrapolation using our approach. Although data on travel speeds used in this study were based on studies in Africa and travel-time modeling using friction surfaces is an established method in global health accessibility research, we acknowledge the absence of real-time GPS tracking of patient journeys and that our study may not fully reflect the realities of local travel in Makueni. The analysis did not examine the incidence of exposure to rabies across the study region. Accounting for variation in risk of exposure may improve the optimization of facilities that should provide PEP to increase access. Although our analysis assumes that geographical time to health facilities is the primary determinant of access, this may also be further influenced by factors such as socio-economic status, risk perception of the individuals, and levels of awareness and knowledge about rabies in the community.

Edge effects can lead to over-estimating the proportion of the population outside of a defined travel time to health facilities (people close to the administrative boundaries may seek health services in facilities in a neighboring region), but we expect this to have a minimal effect on our estimates. This is because rabies PEP is provided at a cost to the patient and Makueni is the only county in the region to have subsidized access to healthcare through the universal health coverage program that included rabies PEP. It is also worth noting that the contact tracing data could be biased and only show the patients living within facilities that stock and provide PEP. These data might presumably show more patients (likely at lower risk) seek PEP if they live close to facilities, whereas our data might precisely miss those who did not seek care due to geographical barriers. Contact tracing also defined probable rabid dogs based on clinical history and outcome but we do not have laboratory confirmation for the animals involved in biting incidents.

Taken together, this analysis provides potential guidance for expanding access to rabies PEP in a region implementing rabies elimination programs. We take the realistic view that facilities already providing PEP would be unlikely to discontinue providing PEP even if not optimally located. Given that rabies is not a single-dose vaccine and that the Kenyan regimen requires five doses, a patient living within 10 min of a facility would spend approximately 50 min cumulatively completing the full course. In contrast, those living more than 60 min away would spend at least five-h traveling, excluding waiting times and costs. These substantial time burdens highlight the continued need to minimize travel distance and time to ensure equitable access to this life-saving vaccine. Overall, our findings suggest that increased geographic distance and prolonged travel time substantially reduce the likelihood that bite victims will seek and complete PEP. Strategic expansion of PEP facilities, guided by travel-time modeling such as that presented here, can significantly improve accessibility within recommended time thresholds and help prevent human rabies deaths in rural, endemic settings.

## Data Availability

The datasets presented in this study can be found in online repositories. The names of the repository/repositories and accession number(s) can be found below: the R code and Makueni facility locations datasets used for the analysis are available at: https://github.com/CEMA-DataScience/rabies_PEP_access/tree/main/Makueni.
